# Quantifying spinal cord cross-sectional area in HTLV-1 associated myelopathy/tropical spastic paraparesis (HAM/TSP)

**DOI:** 10.1186/1742-4690-11-S1-P22

**Published:** 2014-01-07

**Authors:** Winston Liu, Anshika Bakshi, Raya Massoud, Giovanna S Brunetto, Daniel S Reich, Govind Nair, Steven Jacobson

**Affiliations:** 1 Neuroimmunology Branch, National Institute of Neurological Disorders and Stroke, NIH, Bethesda, MD, USA; 2 Fischell Department of Bioengineering, University of Maryland, College Park, MD, USA

## 

The inflammation and subsequent atrophy of the spinal cord are thought to underlie the debilitating symptoms of HAM/TSP. Although spinal cord atrophy can be qualitatively detected on routine clinical MRI, a robust and sensitive method to quantify changes in spinal cord size might capture disease processes. We have developed a novel and fast algorithm to determine the average cross-sectional area in the cervical (c-spine) and thoracic (t-spine) spinal cords by tracing contours perpendicular to the edge in T1-weighted MRI images. The cross-sectional areas in the c- and t-spines were determined in 11 HAM/TSP, 10 multiple sclerosis (MS), and 7 healthy control subjects. Average cross-sectional area in both the t-spine and c-spine were significantly lower in HAM/TSP patients (t-spine: 26.1 ± 5.0 mm^2^; c-spine: 51.9 ± 6.8 mm^2^) as compared to MS patients (t-spine: 39.7 ± 8.9 mm^2^, p=0.0004; c-spine: 68.9 ± 11.6 mm^2^, p=0.0005) and healthy controls (t-spine: 43.5 ± 5.5 mm2, p<0.0001; c-spine: 84.2 ± 10.9 mm2, p<0.0001). Appreciating the small sample size, t-spine cross-sectional area correlated with blood serum proviral loads (r=-0.62, p=0.04, n=9) and with clinical disability measured by EDSS and IPEC. These results suggest that the pattern of spinal cord tissue damage is specific to the underlying inflammatory disease, a finding that has direct implications for the use of average cross-sectional spinal cord area as a surrogate end point for clinical trials.

